# Diet-induced hyperoxaluria: A case based mini-review 

**DOI:** 10.5414/CNCS111505

**Published:** 2024-10-02

**Authors:** Aman Pal, Emmanuel Aydin-Ghormoz, Andrea Lightle, Geovani Faddoul

**Affiliations:** 1 Department of Medicine,; 2 Department of Medicine, Division of Nephrology and Hypertension Care, and; 3 Department of Pathology and Laboratory Medicine, Albany Medical Center, Albany, NY, USA

**Keywords:** oxalate nephropathy, acute kidney injury, nutrition and metabolism

## Abstract

Introduction: Oxalate nephropathy (ON) is a rare condition involving the precipitation of calcium oxalate crystals within the nephrons. Primary hyperoxaluria involves enzymatic defects in the metabolism of glyoxylate, while secondary hyperoxaluria includes dietary and malabsorption-related etiologies. Case presentation: We discuss the case of a White male in his 80s who presented to the hospital with acute kidney injury on chronic kidney disease stage 4 in the setting of a new antibiotic prescription. Creatinine had increased to 4.2 mg/dL from a baseline of 2.2 mg/dL, with no etiology identified on urinalysis or renal ultrasound. Renal biopsy then revealed an acute tubular injury with intraluminal calcium oxalate crystals deposits, confirming a diagnosis of ON. Discussion: A detailed history revealed an excessive dietary intake of oxalate-rich foods, including nuts, and daily ingestion of 2 g of vitamin C. The patient was counselled on adjusting his diet and stopping vitamin C supplementation, which led his creatinine to return close to baseline 2 months post-discharge. Conclusion: Thorough history-taking enables early recognition and timely interventions to possibly avoid hyperoxaluria from progressing to end-stage kidney disease (ESRD).

## Background 

Since the origins of medicine in the 5^th^ century BC, generations have adopted a systematic approach towards patient care by demonstrating a structure in history taking and displaying skills in a physical examination. Key components from these minimally invasive assessments allow physicians to gather information and form a diagnosis to pursue a course of treatment in the best interest of the patient [1]. However, over the years, with the evolution of technology, rapid access to resources, crunching time limits, and increasing prevalence of the ill, the reliance on investigations has increased, and the depth of histories has decreased. Our case highlights this topic as diagnosis of oxalate nephropathy (ON) secondary to excessive dietary intake of nuts and vitamin C can be elicited by taking a thorough diet and supplementals history. Additionally, our case emphasizes how timely diagnosis can possibly avoid progression to end-stage kidney disease (ESRD). 

## Case presentation 

A White male in his 80s presented for evaluation of an acute kidney injury (AKI) superimposed on a known stage 4 chronic kidney disease (CKD). His medical history included bladder cancer status post cystectomy and neobladder 20 years ago with need for twice-daily self-catheterization, complicated by a colovesical fistula and recurrent urinary tract infections (UTIs). He was being followed by nephrology, and his CKD was thought to be related, in part, to reflux nephropathy secondary to his neobladder. 

A few weeks prior to his presentation, the patient described an incident where he felt a sharp burning pain on the lateral aspect of his right lower leg. He reached down and pulled away a hard object from his skin, which he believed to be an insect, but was unable to visualize anything. A few days after this questionable bite, the patient began experiencing fatigue and malaise with subjective fevers. He visited his primary care provider (PCP) who prescribed him a 1-week course of doxycycline 100 mg twice daily for tick-borne infection treatment, and his symptoms resolved within the week. The patient returned to his PCP for routine blood work, and he was noted to have a creatinine rise to 4.2 mg/dL from a baseline of 2.2 mg/dL. However, his PCR tests for *Anaplasma phagocytophilum*, *Ehrlichia chaffeensis*, and *Babesia Microti* were all negative. He denied any symptoms at this time and denied any non-steroidal anti-inflammatory drugs (NSAID) use. His medications at the time included aspirin, atorvastatin, losartan, sertraline, and sodium bicarbonate. During this evaluation, the patient failed to disclose that he was taking ascorbic acid for preventative measures. His family history was non-contributory. He was a former smoker with a 20-pack year history, occasionally drank alcohol, and denied any illicit drug use. 

He was referred to the emergency department by his PCP for further investigation and management of his AKI. 

## Investigations 

On admission, the patient’s bedside urinalysis revealed 1+ protein, 3+ leukocyte esterase, negative nitrites, 1+ hemoglobin, 2 – 5/HPF white blood cells, slight bacteria, and positive eosinophils. Initial bloodwork with complete blood count and complete metabolic panel was remarkable only for the rise in creatinine to 4.2 mg/dL from baseline of 2.2 mg/dL. Tick-borne panel and blood eosinophil levels were non-contributory. Renal ultrasound demonstrated an increased cortical echotexture consistent with medical renal disease. 

After remaining asymptomatic and vitally stable, the patient was discharged home and advised to follow-up in the nephrology outpatient clinic at the end of the week. By this time, his renal function had not improved, and this prompted a renal biopsy to narrow down the differential diagnoses. Possibilities included drug-induced acute interstitial nephritis (DI-AIN), which would warrant steroid use, an acute tubular injury, or an intraglomerular disease. To note, this was the patient’s first documented biopsy as his pre-existing CKD was a clinical diagnosis likely in the setting of hypertension and chronic reflux nephropathy with recurrent UTI. 

**Figure 1.**
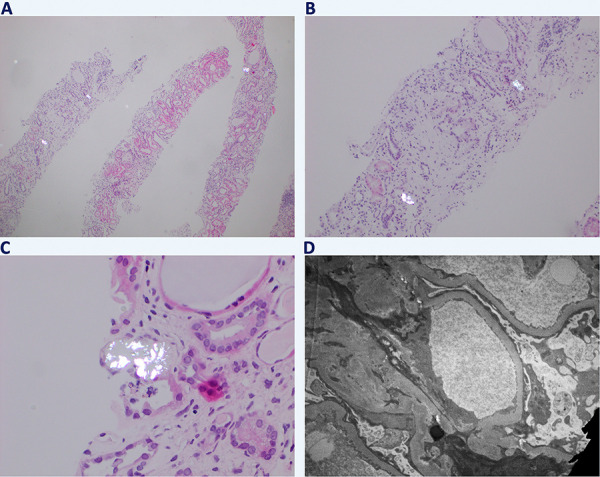
Birefringent intraluminal calcium oxalate crystals are demonstrated by polarization of H & E-stained slides at magnifications including × 40 (A), × 100 (B), and × 400 (C). Final image (D) demonstrates the 40%-foot effacement process seen under electron microscopy.

The patient underwent a biopsy of his right kidney which revealed acute tubular injury with calcium oxalate crystals, severe arteriosclerosis, moderate arteriolar hyalinosis, 40% foot effacement, and moderate interstitial fibrosis and tubular atrophy. (Figure 1. biopsy) 

## Treatment 

The patient was updated on the results of his biopsy and that the diagnosis of ON was considered the etiology of his AKI. Upon further questioning, it was discovered that the patient was taking 2 g of vitamin C daily for several years to acidify his urine for infections and had recently considerably increased his intake of nuts over the preceding months serving as a healthy snack. Given the high intake of oxalate-rich foods, the patient was advised to cut his nut intake from his diet and increase his daily fluid intake. He stopped his vitamin C, and his sodium bicarbonate dose (given for metabolic acidosis) was increased to alkalize his urine. 

## Outcome and follow-up 

**Figure 2.**
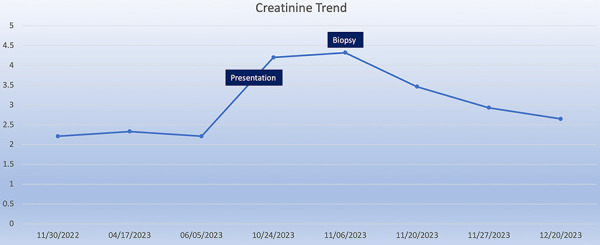
Creatinine trend overtime in mg/dL with key events including presentation of acute kidney injury, biopsy, and post-intervention results.

The patient’s creatinine gradually improved to 2.65 mg/dL (Figure 2), and it remained stable up to 2 months later. Since stopping his supplemental vitamin C and nuts from his diet, the patient has not reported any symptoms and continues to produce adequate urine. With the assistance of his PCP, this patient’s AKI was detected at an early stage, and a biopsy was performed in a timely manner to discern its reversible etiology and prevent progression into ESKD. 

Another interesting element of this case is that during the initial work-up of his CKD 2 years prior to current presentation, the patient underwent a Renasight genetic test. This came back positive as carrier for NPSH2 gene mutation, a gene which is responsible for the production of podocin and can be pathogenic even in its recessive form. This mutation could have played a role in the 40% foot effacement process seen under the electron microscopy in Figure 1. Although there are no relationships with oxalate formation, this factor could independently play a role in the development of ESKD. 

## Discussion 

In our case, the leading differential for the rapid rise in creatinine was a DI-AIN, given the patient’s recent exposure to doxycycline and the non-specific findings of leukocytes and eosinophils in the urine sediment. Although DI-AIN accounts for nearly 20% of cases of unexplained AKI, there has been no conclusive evidence to suggest an association between doxycycline and AIN aside from a few isolated cases that demonstrated resolution of the AKI after withdrawing from doxycycline [2, 3, 4]. Diagnosing AIN remains challenging due to the poor sensitivity and specificity of current diagnostic tests, such as urine eosinophils and leukocytes [5]. Our patient’s creatinine remained elevated despite removal of the offending agent, and a subsequent kidney biopsy, typically definitive for diagnosis, did not show AIN. 

**Figure 3.**
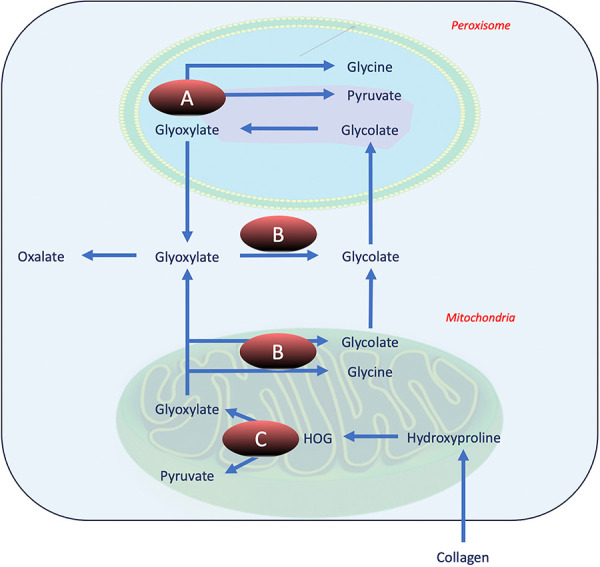
Illustration of enzymatic defects in primary hyperoxaluria. A: Alanine glyoxylate aminotransferase (AGT1) defect seen in primary hyperoxaluria (PH) type 1. B: Glyoxylate/hydroxypyruvate reductase (GRHPR) defect seen in PH2. C: 4-hydroxy-2-oxoglutarate aldolase (HOGA) defect seen in PH3.

The biopsy revealed acute tubular injury with calcium oxalate crystals. The etiologies of ON, which will be reviewed briefly, are classified into primary and secondary hyperoxaluria. Primary hyperoxaluria (PH) is characterized by genetic defects involved in the oxalate metabolism, and it can be classified into three subtypes, PH1, PH2, and PH3 (Figure 3) [6]. After learning about our patient’s Renasight genetic testing, we retrospectively checked the results, and our patient did not have mutations in the AGXT, GRHPR, or HOGA genes, making PH unlikely. 

**Figure 4.**
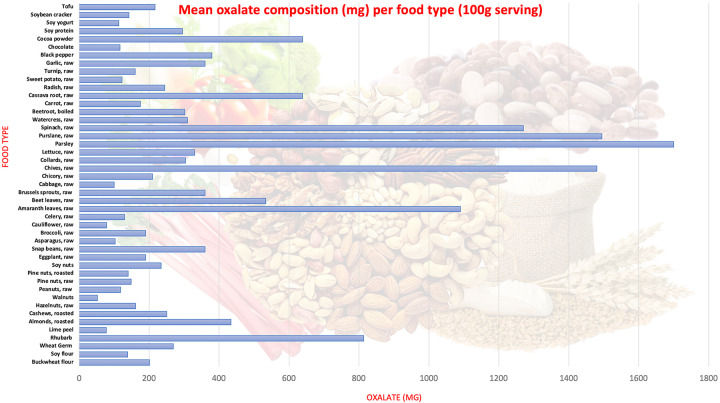
Mean oxalate composition (mg) per food type (100 g serving).

Secondary causes of hyperoxaluria consist of excessive intake of oxalate-rich foods, intestinal malabsorption, and altered enteric flora. Nasr et al.’s [7] retrospective analysis of 113 patients with secondary ON found that 60% of cases were attributed to enteric hyperoxaluria, 23% to high oxalate diet such as vitamin C, and 17% were idiopathic. Similarly, in a meta-analysis by Lumlertgul et al. [8], which analyzed 57 case reports and 10 case series of secondary ON, fat malabsorption was found to contribute to 88% of cases, while dietary consumption contributed to 20%. Diet-induced hyperoxaluria is a well-documented topic, as evidenced by numerous case reports detailing the overconsumption of foods such as spinach, cashew nuts, peanuts, and vitamin C [9]. Our case is different in that there was no single-inciting factor that triggered the condition; instead, it was the cumulative effect of various dietary products, namely almonds, walnuts, peanuts, pine nuts, and vitamin C. The composition of oxalate in each of the nuts is described in Figure 4, and our patient consumed ~ 30 g of these nuts in his oatmeal up to several times a day. In a 30-g serving, the amount of oxalate equates to 130 mg in almonds, 36 mg in peanuts, 60 mg in pine nuts, and 16 mg in walnuts, for an estimated total of 242 mg per oatmeal serving. Additionally, our patient consumed 2,000 mg of vitamin C every day, which is well-above the daily recommended amount of 90 mg a day for men. While our patient did not demonstrate any active bowel disease, his recent ailment may have resulted in decreased water intake and eventual decrease in urine volume. All these factors played an integral part in the development of his AKI, and it remained prudent to collect a thorough dietary history after his biopsy to reverse the etiology and prevent progression into ESKD. 

Oxalate is abundant in both animal and plant sources, with the highest concentrations found in foods like spinach, tofu, nuts, plums, chocolate, beetroot, rhubarb, and strawberries (Figure 4) [10, 11, 12, 13, 14]. However, despite their high oxalate content, these foods are rarely the sole cause of ON due to unlikely consumption of large quantities individually. More common culprits identified in studies include peanuts, almonds, cashew nuts, soy protein, and rhubarb. Dietary oxalate levels can vary widely, ranging from 44 to 351 mg/day on a standard American diet, complicating its correlation with calcium oxalate stone formation [15]. Current literature has shown a non-linear relationship between dietary oxalate intake and urinary oxalate levels, with an approximate 2.7-mg increase in urinary oxalate per 100 mg of dietary oxalate on a 1,000 mg/day calcium diet [16]. Ascorbic acid (vitamin C), a readily available over-the-counter supplement, is a well-established precursor of oxalate and can acidify the urine to precipitate the formation of oxalate crystals. While beneficial, it can increase urinary oxalate levels significantly, with a dose of 1,000 mg shown to increase urinary oxalate up to 13 mg/day [17]. Hence, whether the triggers are certain food products or the use of supplements, consuming excessive amount of oxalate could result in a urinary oxalate level above the appreciated threshold of 44 mg/day for association with calcium oxalate stones [17]. 

Dietary intake contributes to ~ 50% of the oxalate excreted in urine, but its impact is not linear due to factors affecting oxalate bioavailability in the gut [15]. Absorption rates can vary widely, from 10 to 72%, influenced by factors such as food processing, cooking methods, and the presence of *Oxalobacter formigenes*, a bacterium that consumes oxalate [18]. Certain conditions like inflammatory bowel disease, chronic pancreatitis, and bariatric surgery can increase oxalate absorption by altering gut physiology [19]. In these cases, fatty acids may bind calcium instead of oxalate, allowing more oxalate to be absorbed into the bloodstream [19]. Chronic volume contraction due to fluid and salt loss in the gut can also lead to decreased urine volume and increase the risk of oxalate crystallization and stone formation [19]. Conversely, calcium and magnesium supplementation, as well as certain cooking methods and food compositions, can decrease oxalate bioavailability, thereby reducing urinary oxalate excretion [18, 20]. 

## Conclusion 

Our case highlights the diagnostic complexity of ON during an unexplained AKI and emphasizes the critical role of comprehensive history-taking. Early identification of dietary factors and supplements is crucial in possibly preventing invasive procedures such as a renal biopsy and the advancement to ESKD in patients with underlying CKD. 

## Contributions 

AP, EAG, AL, and GF participated in data collection. AP and wrote the manuscript. EAG and GF corrected the manuscript. GF supervised the project. AP, EAG, AL, and GF take responsibility that this study has been reported honestly, accurately and transparently, and accept accountability for the overall work by ensuring that questions pertaining to the accuracy or integrity of any portion of the work are appropriately investigated and resolved. 

## Consent 

Written consent was obtained from the patient. 

## Data availability 

Access to data is permitted with the authors’ permission. 

## Funding 

None**.**

## Conflict of interest 

The authors declare that they have no conflict of interest.
